# Value of the Electronic Medical Record for Hospital Care: Update From the Literature

**DOI:** 10.2196/26323

**Published:** 2021-12-23

**Authors:** Aykut Uslu, Jürgen Stausberg

**Affiliations:** 1 USLU Medizininformatik Düsseldorf Germany; 2 Institute for Medical Informatics, Biometry and Epidemiology University Hospital Essen University Duisburg-Essen Essen Germany

**Keywords:** cost analysis, costs and cost analyses, economic advantage, electronic medical records, electronic records, health care, hospitals, medical records systems computerized, quality of health care, secondary data

## Abstract

**Background:**

Electronic records could improve quality and efficiency of health care. National and international bodies propagate this belief worldwide. However, the evidence base concerning the effects and advantages of electronic records is questionable. The outcome of health care systems is influenced by many components, making assertions about specific types of interventions difficult. Moreover, electronic records itself constitute a complex intervention offering several functions with possibly positive as well as negative effects on the outcome of health care systems.

**Objective:**

The aim of this review is to summarize empirical studies about the value of electronic medical records (EMRs) for hospital care published between 2010 and spring 2019.

**Methods:**

The authors adopted their method from a series of literature reviews. The literature search was performed on MEDLINE with “Medical Record System, Computerized” as the essential keyword. The selection process comprised 2 phases looking for a consent of both authors. Starting with 1345 references, 23 were finally included in the review. The evaluation combined a scoring of the studies’ quality, a description of data sources in case of secondary data analyses, and a qualitative assessment of the publications’ conclusions concerning the medical record’s impact on quality and efficiency of health care.

**Results:**

The majority of the studies stemmed from the United States (19/23, 83%). Mostly, the studies used publicly available data (“secondary data studies”; 17/23, 74%). A total of 18 studies analyzed the effect of an EMR on the quality of health care (78%), 16 the effect on the efficiency of health care (70%). The primary data studies achieved a mean score of 4.3 (SD 1.37; theoretical maximum 10); the secondary data studies a mean score of 7.1 (SD 1.26; theoretical maximum 9). From the primary data studies, 2 demonstrated a reduction of costs. There was not one study that failed to demonstrate a positive effect on the quality of health care. Overall, 9/16 respective studies showed a reduction of costs (56%); 14/18 studies showed an increase of health care quality (78%); the remaining 4 studies missed explicit information about the proposed positive effect.

**Conclusions:**

This review revealed a clear evidence about the value of EMRs. In addition to an awesome majority of economic advantages, the review also showed improvements in quality of care by all respective studies. The use of secondary data studies has prevailed over primary data studies in the meantime. Future work could focus on specific aspects of electronic records to guide their implementation and operation.

## Introduction

This review is an update of 2 previous literature analyses on the benefits and costs of electronic medical records (EMRs), based on articles from 1966 to January 2004 [1] and from 2004 to 2010 [2]. Using the same method, this review explores the progress in evidence from empirical studies. The World Health Organization (WHO) has a clear position concerning the evidence for eHealth in general. Already in 2005, the WHO noted, “the potential impact that advances in information and communication technologies could have on health-care delivery...” [3]. Ten years later, the WHO put this straight by stating several advantages of electronic health records (EHRs) in the report of the third global survey on eHealth, which was produced by the Global Observatory for eHealth [4]:

- EHRs improve the quality, accuracy, and timeliness of patient information at the point of care.
- EHRs provide insights into health care costs, utilization, and outcomes.
- EHRs promote quality of care, reduce costs, support patient mobility, increase reliability of information, and provide access to patient information to multiple health care providers.
- Analyses from EHR data can highlight areas of concern and health services delivery.

The latter is emphasized in the current European digital strategy for data by creating a common European health data space that ensures interoperability of health data and in which every citizen has secure access to his or her EHR [5]. Consequently, many states adopted these visions and implemented national strategies for eHealth in general and for the EHR in particular (see [6] for an overview of Europe or [7] for country profiles from the Global Observatory for eHealth). In the United States, the meaningful use of health care information technology (IT) was fostered by the implementation of EHRs for all citizens until 2014 through the Health Information Technology for Economic and Clinical Health (HITECH) Act [8,9]. HITECH was successful, increasing the hospitals’ adoption rate of a basic EHR from 9.4% in 2008 and 15.6% in 2010 to 97% in 2014 [10]. In Germany, the Patient Data Protection Act “obliges the public sickness funds to offer their clients an electronic patient record (EPR) not later than 1 January 2021” [11]. Furthermore, physician practices and hospitals are requested to support and to use the EPR based on the legal basis of an informed consent by the patients. In 2017, half of the German hospitals quoted the existence of an institutional electronic record similar to the situation in Austria [12]. Only the Swiss hospitals reported a higher proportion with 78%, a statistically significant difference to Germany.

EHRs will offer basic values by providing “the right information at the right time in the right place” [13]. This aim is achieved by improving the traditional function of patient records to store information relevant to the care. However, EHRs should additionally guide the process of clinical problem solving and should support clinical decision making [14]. In 1991, the Institute of Medicine (IOM) listed 4 ways to positively influence quality of care [14]: (1) improving quality of and access to clinical data, (2) integrating information over time and settings, (3) making knowledge available, and (4) providing decision support. Looking at costs, the IOM expected positive effects in 3 ways: (1) reducing unnecessary tests and services, (2) reducing administrative costs, and (3) increasing the productivity of health care professionals.

One might argue that a further discussion about the proposed value of an EMR is needless because of its nearly complete implementation. Nobody will vote for a fallback to paper. However, the implementation does not guarantee a positive perception by the users. In a recent survey including 208 physicians from 3 Norway hospitals [15], 72% of the physicians reported interrupted or delayed work at least once a week because the EHR hangs or crashes, and 53% of the physicians indicated that the EHR is cumbersome to use and adds to their workload. These results demonstrate a reasonable room for improvements, besides noncontroversial advantages that were reported in the study from Norway. Even if up-to-date health care cannot be imagined without an EMR, an ongoing evaluation of its advantages and disadvantages is a prerequisite for a well-considered further development and adjustment. In our sequence of literature reviews, we put the ultimate goals of health care in the middle, to provide a high level of care for reasonable costs in terms of effectiveness and efficiency [16]. Furthermore, the series of reviews allows a monitoring of the EMR’s value over time by preserving the criteria for the selection and the appraisal of the included studies. The research questions were twofold. What is the effect of EMRs on the quality of inpatient care? What is the effect of EMRs on the costs for inpatient care?

## Methods

### Terminology of Electronic Records in Health Care

Concepts and terms denoting electronic records in health care are still not unambiguously defined [17]. Differences and similarities of “electronic medical records,” “electronic patient records,” and “electronic health records” are a matter of a long-lasting debate. In our reviews, we focused on electronic records used by health professionals and administrative staff for inpatient care, including, for example, physicians, nurses, radiologists, pharmacists, laboratory technicians, and radiographers [17]. Those records must not necessarily follow a patient lifelong. Therefore, we adopted the definition of an EMR by Waegemann [18]: an EMR is a “computer-stored collection of health information about a person, linked by a person identifier”, with the application environment being a hospital and including any care delivery being the full responsibility of the health care provider.

### Search Strategy

The literature search was performed between March 10, 2019, and April 2, 2019, using MEDLINE. MEDLINE was accessed via PubMed [19]. The keyword “Medical Records Systems, Computerized” from the MeSH was separately combined with the MESH terms “technology assessment, biomedical”, “costs and cost analysis”, “health care costs”, “cost savings”, “cost effectiveness”, “cost benefit”, “cost analysis”, “benefits and costs”, “quality of health care”, “outcome study”, “outcome assessment, patient”, and “critical care outcomes”. Additionally, January 1, 2010, was defined as the earliest date of publication. After an exclusion of duplicates, interactive tutorials and reviews, and a restriction to the languages German and English, 1345 references remained.

### Study Selection

Using titles and abstracts, both authors independently reviewed the 1345 literature references regarding the existence of an EMR, the application of an EMR in inpatient care, and an empirical analysis of benefits or costs. Explicitly excluded were studies in physician offices or about ambulatory care provided by hospitals, studies about picture archiving and communication systems, and studies about systems for computerized physician order entry (CPOE). The rating comprised the categories accept, refuse, and unclear. References rated as accept/accept and accept/unclear were qualified, references rated as refuse/refuse and refuse/unclear were rejected. References rated as accept/refuse or unclear/unclear were discussed and a final decision was reached based on a consensus. Herewith, 84 publications were qualified for the further evaluation (6.25%). From these, full texts of 79 papers could be obtained; for 5 papers, this was not possible. Textbox 1 shows the inclusion and exclusion criteria of both stages.

**Inclusion and exclusion criteria.**

**Inclusion criteria**

- Acute care hospital
- Inpatient care
- Electronic medical record
- Empirical result
- Statement about costs
- Statement about benefits

**Exclusion criteria**

- Physician office
- Ambulatory care
- Picture archiving and communication system
- System for computerized physician order entry

**Figure 1:**
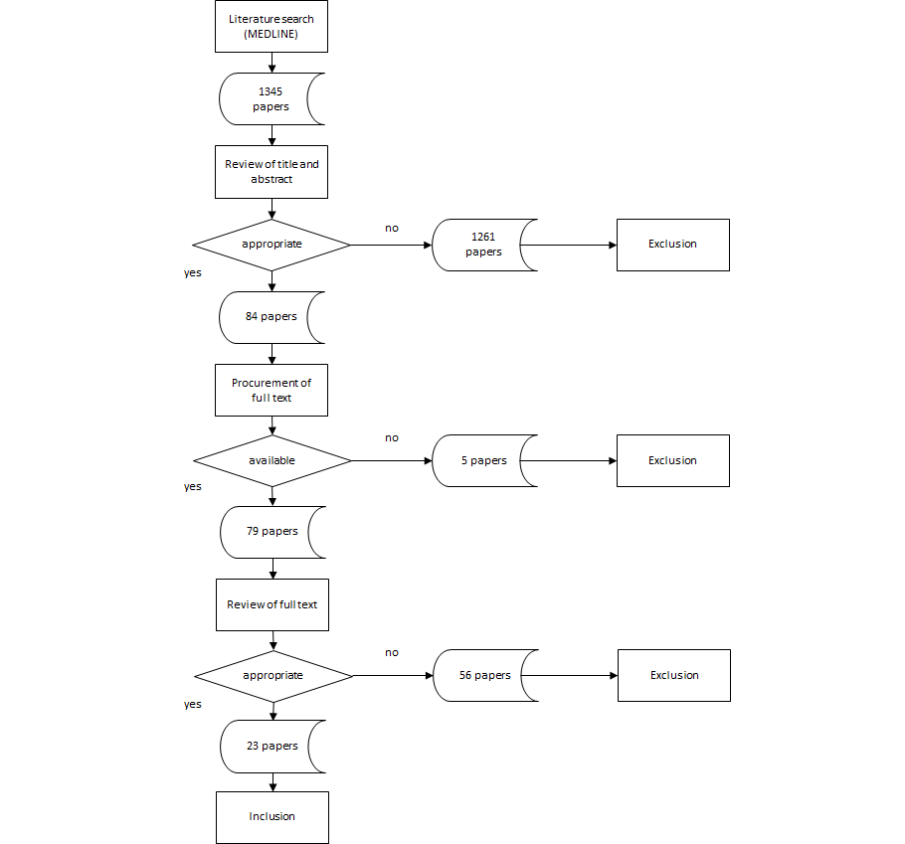
Selection and review process.

Both authors again carried out the evaluation of the remaining 79 publications independently. This time, the evaluation was based on the full texts of the references. Both authors looked at concrete statements on benefits and costs, and gave a final recommendation about the inclusion into the review. References were finally included if they reached 2 or 3 positive votes from both authors (16/79 references, 20%). References were finally excluded if neither authors gave at least two positive votes (43/79 references, 54%). The remaining 20 references were discussed to reach a consensus about their inclusion for the review (25% from 79 references). Overall, the selection process produced 23 relevant studies that ultimately formed the subject of the detailed analysis, being 1.71% from the initially identified references (N=1345; Figure 1).

Interrater reliability during study selection was verified by calculating Cohen κ. In the first evaluation level based on titles and abstracts, the κ value was 0.185, indicating a slight agreement between the reviewers according to the interpretation of Landis and Koch [20] (Table 1). In the second evaluation level of full texts, the κ value was 0.428, indicating a moderate agreement. The interrater reliability was comparable to the previous reviews.

**Table 1:** Interpretation of κ values [20].

κ value	Level of agreement
<0.00	Poor
0.00-0.20	Slight
0.21-0.40	Fair
0.41-0.60	Moderate
0.61-0.80	Substantial
0.81-1.00	Almost perfect

### Study Evaluation

For a semiquantitative evaluation of the studies, a catalog of criteria was drawn up focusing on the study design, the formal quality of the publication, the number of users included, the study duration, and the use of statistical tests. Each aspect of a study was rated 2, 1, or 0 points, with 2 being the best score for a study. Missing information was rated 0 points. The maximum number of points that could be achieved was therefore 10. In addition, the studies were described with regard to their origin, their application scenarios, and their target values. The approach proposed by Johnston et al [21] was adopted as basis for the evaluation method. The definition of the criteria was partly different between studies collecting primary data and studies analyzing existing, secondary data. The definition was carried out as described below.

### Study Design

The assessment of the study design was based on the classification depicted in Table 2, according to Roine et al [22]. Different types of studies were ranked from 1 to 9 concerning the evidence hierarchy. The first stage, meta-analyses from randomized, controlled studies, was not a component of the inclusion criteria. The remaining study types were combined into the following 3 groups: randomized controlled studies (evidence stages 2 and 3); nonrandomized controlled studies (evidence stages 4, 5, 6, and 7); and uncontrolled clinical series, descriptive studies, consensus methods, application observations, and empirical reports (evidence stages 8 and 9). Studies in the first group received 2 points, studies in the second group 1 point, and the remaining studies 0 points. According to the proposal of Nathan and Gorman [23], all secondary data analyses were assigned to evidence stage 7 of Table 2 and were uniformly assigned 1 point. Therefore, the maximum number of points was reduced to 9 for those studies.

**Table 2:** Classification of study designs [22].

Evidence stage	Study design
1	Meta-analyses of randomized controlled trials
2	Large-sample randomized controlled trials
3	Small-sample randomized controlled trials
4	Nonrandomized controlled prospective studies
5	Nonrandomized controlled retrospective trials
6	Cohort studies
7	Case–control studies
8	Noncontrolled clinical series, descriptive studies, consensus methods
9	Anecdotes or case reports

### Formal Quality of Publication

The publication should follow the international standard structure of scientific articles, that is, authors’ names and affiliations on the title page, abstract, introduction, material and methods, results, discussion, conclusions, and references. For a publication in full compliance with this structure, 2 points were assigned; if the article provided a separate introduction and an explicit naming of authors and the medical environment, 1 point was assigned; otherwise, 0 points were given.

### Number of Users

The number of EMR users can affect the reliability and the generalizability of the results. Therefore, 2 points were given for studies based on primary data with 20 or more users, 1 point for 6-19 users, and 0 points for less than 6 users or if no number of users was specified. For studies analyzing secondary data, the number of hospitals included was scored as follows: 2 points for a hospital number of 2000 and above; 1 point for a hospital number of 500 to 1999; and no points for a hospital number of 0-499 or missing information.

### Implementation Duration

Primary data studies implemented for at least one year received 2 points, 1 point was given for a half to less than 1 year, and 0 points for less than a half year. For the secondary data studies, the evaluation periods were scored as follows: 2 points were awarded to a study for an evaluation period of 3 years or longer, 1 point for a period of 1 or 2 years, and no points for an implementation period of less than 1 year or in case of missing information.

### Statistical Evaluation

Assessment and evaluation of scientific statements gain in evidential power with inferential statistical statements. Two points were given for studies reporting the result(s) of statistical analyses with full information concerning the level of significance, and 1 point for the description of a statistical test performed without indication of the level of significance. Otherwise, 0 point was given.

## Results

### Origins and Locations of the Studies

The 23 studies selected for the main evaluation [24-46] consisted of 6 primary and 17 secondary data studies (Table 3). Three (7, 18, and 23) of the 6 primary data studies were conducted in the United States (Wisconsin, North Carolina, and Massachusetts), 1 study each was conducted in China (21), Germany (3), and Japan (17). Sixteen secondary data studies originated from the United States, 1 from the Netherlands.

In the secondary data studies, 15 different data sources were used to analyze the issues of treatment quality, costs, and EMR equipment (Table 4). The most frequently used data sources stemmed from the American Hospital Association (AHA; 19 studies), the Healthcare Information and Management Systems Society (HIMSS; 11 studies), and the Centers for Medicare & Medicaid Services (CMS; 9 studies). They were followed by the Hospital Quality Alliance database (HQA), the National Database of Nursing Quality Indicators (NDNQI), and the Office of Statewide Health Planning and Development (OSHPD). The remaining sources were used only in 1 study.

**Table 3:** Characteristics of the included studies.

Study number	Reference	Country	Sample size	Period	Main outcomes
1	Adler-Milstein et al [24]	United States	191 hospitals	2 years	EHR^a^ adoption is associated with better performance in terms of payment and length of stay in well-run institutions. EHR adoption may be associated with worse performance in poorly run institutions.
2	Adler-Milstein et al [25]	United States	2591 hospitals (2011)	4 years	Degree of EHR adoption is positively correlated with process adherence, patient satisfaction, and efficiency.
3	Castellanos et al [26]	Germany	Not indicated	6 years	Small increase in profit in the year after the introduction of the patient data management system.
4	DesRoches et al [27]	United States	3049 hospitals	6 months	Presence of clinical decision support is associated with small quality gains. No relationship between EHR level and overall risk-adjusted length of stay, risk-adjusted 30-day readmission rates, and risk-adjusted inpatient costs.
5	Elnahal et al [28]	United States	3101 hospitals	9 months	Higher rates of adoption of key EHR functions among high-quality hospitals.
6	Encinosa and Bae [29]	United States	2619 hospitals	1 year	EMRs^b^ do not reduce the rate of patient safety events. In case of patient safety events, EMRs reduce deaths, readmissions, and spending.
7	Feblowitz et al [30]	United States	Not indicated	2 years	Length of stay increased after implementation of an electronic documentation. Mean time to disposition for admitted patients remained stable.
8	Furukawa et al [31]	United States	5066 hospitals	10 years	Advanced EMR applications may increase hospital costs and nurse staffing levels, as well as increase complications and decrease mortality for some conditions.
9	Furukawa et al [32]	United States	509 hospitals	5 years	Nurse-sensitive patient outcomes improved. EMR implementation may be associated with reduced demand for nurses.
10	Himmelstein et al [33]	United States	4000 hospitals	6 years	Hospital computerization has not achieved savings on clinical or administrative costs. More computerized hospitals might have a slight quality advantage for some conditions.
11	Jarvis et al [34]	United States	2988 hospitals	1 year	Most advanced EHRs have the greatest payoff in improving clinical process of care scores.
12	Jones et al [35]	United States	6057 hospitals	4 years	Availability of basic EHR is associated with a significant increase in health care quality for heart failure.
13	Joynt et al [36]	United States	1236 hospitals	4 years	Patients with stroke are more likely to receive guideline-driven components of care at hospitals with EHRs. Patients are slightly less likely to have a hospital stay longer than 4 days at hospitals with EHRs.
14	Kazley et al [37]	United States	1000 hospitals	1 year	In hospitals with advanced EHRs, patient costs are less compared with hospitals without advanced EHRs.
15	Lee et al [38]	United States	708 hospitals	8 years	Hospitals adopting EMRs experience shorter length of stay and lower 30-day mortality.
16	McCullough et al [39]	United States	3401 hospitals	4 years	Use of EHRs results in improvements in process-of-care measures for patients with heart failure or pneumonia.
17	Nakagawa et al [40]	Japan	Not indicated	7 years	EMR may decrease medical risks, but profitability does not rise more than the investments.
18	Schenarts et al [41]	United States	Not indicated	40 months	Implementation of the EMR is associated with an improvement in several complications and process measures.
19	Teufel et al [42]	United States	2307 hospitals	1 year	Advanced-stage EMR is associated with greater costs per case.
20	van Poelgeest et al [43]	Netherlands	67 hospitals	1 year	No statistically significant association between a hospital’s EMR adoption and an overall quality or safety performance.
21	Xue et al [44]	China	251 physicians and 298,760 patient visits	5 years	Length of stay declines and mortality rate decreases with EMR. An EMR has no positive effect on patient costs.
22	Yanamadala et al [45]	United States	448,767 patients	1 year	Patients at hospitals with full EHR have the lowest rates of inpatient mortality, readmissions, and patient safety indicators.
23	Zlabek et al [46]	United States	Not indicated	Not indicated	Implementation of an inpatient EHR results in a rapid improvement in measures of cost of care.

^a^EHR: electronic health record.

^b^EMR: electronic medical record.

**Table 4:** Sources used by the secondary data studies.

Study number	Source (included years)			
	Quality	Costs	Electronic medical record	Other
1	AHA^a^ (2009)	AHA (2009)	AHA IT Supplement (2009)	World Management Survey (2009)
2	AHA (2009-2012) CMS’s^b^ Hospital Compare (2009-2012)	CMS’ EHR^c^ Incentive Program reports (2009-2012)	AHA IT Supplement (2008-2011) CMS’ EHR Incentive Program reports (2009-2012)	AHA annual survey (2008-2011)
4	AHA (2008) HQA^d^ database (2009)	AHA (2008) Medicare Provider Analysis and Review (2006)	AHA IT Supplement (2008)	
5	HQA database (2006)		AHA IT Supplement (2009)	
6	MarketScan Commercial Claims and Encounter Database (2007) AHA (2007)	MarketScan Commercial Claims and Encounter Database (2007) AHA (2007)	AHA (2007)	
8	OSHPD^e^ (1998-2007)	OSHPD (1998-2007)	HIMSS^f^ (1998-2007)	OSHPD (1998-2007)
9	NDNQI^g^ (2004-2008)	NDNQI (2004-2008)	HIMSS (2004-2008)	
10	Dartmouth Health Atlas (2008)	The Medicare Cost Reports	HIMSS (2003-2007)	
11	AHA (2008-2010)	CMS	HIMSS (2012?)	
12	AHA (2004-2007)		HIMSS (2003-2006)	
13	AHA (2007-2010)	AHA (2007-2010)	GWTG-Stroke^h^ (2007-2010), linked with the AHA annual survey	
14		NIS^i^ (2009)	HIMSS (2009)	
15	MEDPAR^j^ (2000-2007)		HIMSS (2000-2007)	
16	AHA (2004-2007)	CMS (2004-2007)	HIMSS (2004-2007)	
19		HCUP KID^k^ (2009)	HIMSS (2009)	
20	EMRAM^l^ (2014)		EMRAM (2014)	
22	HCUP, SID^m^ (2011)		AHA annual survey (2008, 2011)	

^a^AHA: American Hospital Association.

^b^CMS: Centers for Medicare & Medicaid Services.

^c^EHR: electronic health record.

^d^HQA: Hospital Quality Alliance database.

^e^OSHPD: Office of Statewide Health Planning and Development.

^f^HIMSS: Healthcare Information and Management Systems Society.

^g^NDNQI: National Database of Nursing Quality Indicators.

^h^GWTG-Stroke: Get With the Guidelines-Stroke.

^i^NIS: nursing information system.

^j^MEDPAR: Medicare Provider Analysis and Review

^k^HCUP KID: Healthcare Cost and Utilization Project Kids Inpatient Data.

^l^EMRAM: HIMSS Analytics EMR Adoption Model.

^m^SID: State Inpatient Databases.

### Methodical Quality

The results of the semiqualitative assessment are presented in Table 5 and Multimedia Appendix 1. In the evaluation of the primary data studies, 2 (18 and 21) publications achieved a score of 6 points, 3 (3, 7, and 17) scored 4, and 1 (23) achieved only 2 points. No primary data study scored 0, 1, 3, 5, and 7-10 points. While in the secondary data studies 2 papers (2 and 10) achieved the maximal score of 9 points, another 4 (9, 13, 15, and 16) scored 8, 7 (4-6, 8, 11, 12, and 19) scored 7, 2 (1 and 14) scored 6, 1 (22) scored 5, and 1 (20) scored 4. No secondary data study scored 0-3 and 10 points. A total of 18 of the 23 studies scored 5 and more points (78%), while 5 remained below this score (22%). Only 2/6 (33%) primary data studies achieved 5 points or more. By contrast, 16/17 (94%) secondary data studies achieved a score of 5 points or more.

Two (1 and 19) of the primary data studies were randomized controlled trials; one (4) was a nonrandomized controlled trial; the remaining 3 belonged to a lower evidence stage. By definition, the 17 secondary studies were all assigned to evidence level 7. Fifteen (1-6, 9-11, 13-16, 19, and 20) studies followed the internationally accepted structure of scientific articles. The remaining 8 studies (7, 8, 12, 17, 18, and 21-23) lacked any formal structure.

Three (17, 18, and 21) of the 6 primary data studies had a user population of at least 20 or more. The remaining three (3, 7, and 23) did not provide any information. Five (3, 7, 17, 18, and 21) primary data studies had an implementation period of at least one year, 1 (23) less than 6 months. Eleven (2, 4-6, 8, 10-12, 16, 19, and 22) of the 17 secondary studies included at least 2000 hospitals, 4 (9, 13-15) 500 to less than 2000 hospitals, and 2 (1 and 20) less than 500 hospitals. Eight (2, 8-10, 12, 13, 15, and 16) of the secondary data studies analyzed data from at least three years, 1 (1) from 1 or 2 years, and 8 (4-6, 11, 14, 19, 20 and 22) from less than 1 year.

Nineteen (1, 2, 4-15, 18, 19, and 21-23) of the 23 studies supported the value of their results by statistical tests with full information on the level of significance. Two (16 and 20) studies stated that they had performed statistical tests but did not name them, and 2 (3 and 17) studies did not provide any information on them.

**Table 5:** Final score and conclusions of the included studies.

Study number	Reference	Data source	Final score	Cost reduction	Improvement in quality of care
1	Adler-Milstein et al [24]	S^a^	6	p^b^	p
2	Adler-Milstein et al [25]	S	9	p	p
3	Castellanos et al [26]	P^c^	4	p	n.a.^d^
4	DesRoches et al [27]	S	7	n	x^e^
5	Elnahal et al [28]	S	7	n.a.	p
6	Encinosa and Bae [29]	S	7	p	p
7	Feblowitz et al [30]	P	4	n^f^	x
8	Furukawa et al [31]	S	7	n	p
9	Furukawa et al [32]	S	8	p	p
10	Himmelstein et al [33]	S	9	n	x
11	Jarvis et al [34]	S	7	n.a.	p
12	Jones et al [35]	S	7	n.a.	p
13	Joynt et al [36]	S	8	p	p
14	Kazley et al [37]	S	6	p	n.a.
15	Lee et al [38]	S	8	p	p
16	McCullough et al [39]	S	8	n.a.	p
17	Nakagawa et al [40]	P	4	n	n.a.
18	Schenarts et al [41]	P	6	n.a.	p
19	Teufel et al [42]	S	7	n	n.a.
20	van Poelgeest et al [43]	S	4	n.a.	x
21	Xue et al [44]	P	6	n	p
22	Yanamadala et al [45]	S	5	n.a.	p
23	Zlabek et al [46]	P	2	p	n.a.

^a^S: secondary data studies.

^b^p: positive effect.

^c^P: primary data studies.

^d^n.a.: not assessed.

^e^x: positive effect without specific information.

^f^n: no positive effect.

### Main Subjects

A total of 5 out of the 23 studies (3, 14, 17, 19, and 23) dealt solely with economic aspects of the use of an EMR, 7 (5, 11, 12, 16, 18, 20, and 22) dealt solely with the effects on the quality of care, and 11 studies (1, 2, 4, 6-10, 13, 15, and 21) dealt with both aspects (Tables 3 and 4). Primary data studies and secondary data studies were found in all groups. While 9 (39%) of the 23 studies (1-3, 6, 9, 13-15, and 23) showed an economically positive impact, 7 (30%) (4, 7, 8, 10, 17, 19, and 21) did not reveal monetary advantages due to the use of the EMR. Eighteen studies (1, 2, 4-13, 15, 16, 18, 20-22) looked at the impact of the use of an EMR on the quality of care. All of them (18/23 studies, 78%) found a positive effect. However, 4 (4, 7, 10, and 20) did not provide specific information about it. No study indicated evidence of disadvantages in the quality of treatment from the use of an EMR. Primary data studies and secondary data studies showed similar results.

One of the striking studies, Zlabek et al [46] looked at the effects of an EMR system on selected measures of cost of care and patient safety. They demonstrated the following outcomes (means and % change):

- Laboratory tests per week per hospitalization decreased from 13.9 to 11.4 (18).
- Radiology examinations per hospitalization decreased from 2.06 to 1.93 (6.3).
- Monthly transcription costs declined from US $74,596 to US $18,938 (74.6).
- Numbers of copy paper ordered per month decreased from 1668 to 1224 (26.6).
- Medication errors per 1000 hospital days decreased from 17.9 to 15.4 (14.0), while near misses per 1000 hospital days increased from 9.0 to 12.5 (38.9), and the percentage of medication events that were medication errors decreased from 66.5% to 55.2%.

In a national study about hospital computing and the costs and quality of care, Himmelstein et al [33] analyzed whether highly computerized hospitals had lower costs of care or administration, or better quality. They acquired the following outcomes in their work:

- Higher overall computerization scores correlated weakly with better quality scores for acute myocardial infarction, but not for heart failure, pneumonia, or the 3 conditions combined. In multivariate analyses, more computerized hospitals had a slightly better quality.
- Hospitals on the “Most Wired” list performed not better than others on quality, costs, or administrative costs.
- Hospitals’ administrative costs increased slightly but steadily, from 24.4% in 2003 to 24.9% in 2007. Higher administrative costs weakly predicted higher total Medicare spending, inpatient spending, and outpatient spending.

According to the study performed by Encinosa and Bae [29], many reforms in the Patient Protection and Affordable Care Act (ACA) underlie the use of EMRs to help contain costs. In this regard, the authors found that EMRs do not reduce the rate of patient safety events. However, once an event occurs, EMRs reduce death by 34%, readmissions by 39%, and spending by US $4850 (16%), a cost offset of US $1.75 per US $1 spent on IT capital. Thus, the authors concluded that EMRs contain costs by better coordinating care, a coordination that rescues patients from medical errors once they occur.

The study by Castellanos et al [26] analyzed cost and reimbursement data from a 25-bed intensive care unit at a German university hospital in a retrospective analysis, 3 years before and 3 years after the implementation of a patient data management system (PDMS). Costs and revenues increased continuously over the years. The profit of the investigated intensive care unit was fluctuating over the years and seemingly depending on other factors as well. They found a small increase in profit in the year after the introduction of the PDMS, but not in the following years. Therefore, a clear evidence for cost savings after the introduction of PDMS was not seen.

## Discussion

### Principal Findings

This review is an update of 2 previous analyses on the benefits and costs of EMRs, based on articles from 1966 to January 2004 [1] and 2004 to 2010 [2]. Using the same method, this review explored the progress in evidence from empirical studies. With a total of 19 of the 23 publications selected for evaluation (83%), studies from the United States dominated. Of the remaining 4 studies, 2 were conducted in Europe. Asia was represented by 1 Chinese and Japanese study each. South America, Africa, and Australia were not represented at all. Results of our reviews over the 3 periods showed a number of significant developments (Table 6). For example, the total number of initial hits had almost doubled. While the number of studies relevant to the evaluation remained more or less the same for the first and the current review, the second review produced almost one-third fewer studies. Remarkable in the current review was the predominant use of secondary data studies compared with primary data studies. In this context, highlighting the differences between primary and secondary studies should help to better assess the conclusions drawn from the results. While the primary data studies collected new and yet unexplored data, the secondary data studies used statistical processing of already existing data. In general, secondary data studies do not reach the evidence level of meta-analyses comprising also already existing but initially primary data. The most important advantage of primary data studies is that data can be collected and statistically evaluated in a targeted and problem-oriented manner. Their disadvantage is that specific surveys of patient data are often time-consuming and expensive compared with secondary data studies. Furthermore, in case of complex interventions, as it is the case for EMRs, primary data studies are often not feasible [47]. The advantage of secondary data studies is that comparatively few resources are required to prepare them. Their disadvantage is that the data were not collected specifically to answer the research questions as part of a specifically designed study design.

The annual number of studies on EMRs showed a continuous increase over our 3 review periods (Table 6). The same was true for the annual number of finally included studies. The methodological quality of the studies changed as well. While only 35% of the studies scored more than 5 points in the first review (7/20), 74% of the studies scored more than 5 points in the third review (17/23). Among the finally included studies in the first review, costs were analyzed in 100% of the publications (20/20), with only 20% also focusing on quality of care (4/20). In the second review, both aspects were analyzed in 71% of the publications (5/7). In this review, costs were analyzed in 70% of the publications (16/23), quality of care in 78% (18/23).

The comparison of the 3 periods revealed a twofold shift. On the one hand, the studies’ focus switched from an economical one to a clinical one. The percentage of studies concerned solely with costs decreased from 80% (16/20, 1966-2004) to 14% (1/7, 2002-2010) and 22% (5/23, 2010-2019). On the other hand, the positive effects of EMRs on quality of care became apparent over time. In the first review, none of the 4 studies concerned with quality of care presented well-defined advantages. In this review, this was the case in 14 of 18 studies analyzing the effects of EMRs on quality of care. The reasons for this shift remain speculative. The focus of EMRs might have changed from an administrative one to a patient-oriented one. Technological progress could have helped to achieve the clinical benefits that were an important motivator for the introduction of EMRs even in the early years [48]. In 1997, it was reported that costs remained a significant barrier for EHRs [49]. Now, experiences concerning the introduction, implementation, and an accompanying change management might have better prepared hospitals for the harvesting of clinical benefits and simultaneously for the limiting of additional costs.

**Table 6:** Number of studies considered for the reviews.

Review	Years, n	Hits without duplicates, n	Hits per year, mean	First selection, n	Finally included studies, n	Finally included studies per year, mean
First (1966-2004)	38	588	15.5	117	20	0.5
Second (2004-2010)	6	578	96.3	64	7	1.2
This (2010-2019)	9	1345	149.4	84	23	2.6

### Limitations

The reliability between the 2 authors in selecting the papers was slight in the first phase (κ=0.185) and moderate in the second phase (κ=0.428). Both results were nearly equal compared with the 2 previous reviews, first phase 0.26 (review 1) and 0.192 (review 2), second phase 0.36 (review 1) and 0.399 (review 2). Unfortunately, measures of interrater reliability are usually not presented in systematic reviews. We assume that our results are not inferior in comparison to comparable reviews. The agreement was high in excluding references that do not fulfill the inclusion criteria. Differences occur in the detection of appropriate studies. To avoid the exclusion of false negatives, contrary votes and unclear votes were dissolved in a consensus. However, the extraction of the papers’ main conclusions was a complex process. Misunderstandings and errors in this process cannot be completely ruled out. For example, authors’ conclusions summarized in a paper’s abstract could differ from individual results found in the paper’s main text. The results of univariate and multivariate analyses may not agree and positive effects in one medical condition could be absent in another condition. Therefore, the review’s rating is a pragmatical compromise to reach a meaningful conclusion.

The authors kept the EMR as type of intervention for all 3 reviews and attached great importance to an unaltered approach. This allowed the comparison of results over the whole series of reviews. The decision to maintain the focus on the EMR might be questioned because the literature addresses many different levels of IT used in hospitals. The results are therefore neither tailorable to more detailed types of IT providing only selective functionalities as CPOE nor generalizable to lifelong EPRs or to health information and communication technology overall. Nevertheless, through the clear and persistent focus, the authors gained reliable and valid conclusions beyond transitory trends and fashions.

Furthermore, the series maintained the same set of keywords. The authors could not rule out that newer functionalities of EMRs are not appropriately covered by this set. However, even then, the striking results supporting an indisputable positive effect of EMRs would be an underestimation of the actual situation. It is unlikely that newer functionalities decline the effects of EMRs on quality of care.

The detected studies represent primarily the perspectives of the United States and developed countries. Developed countries have the economic power to implement EMRs and to realize respective evaluation studies. This will not be the case for developing countries. However, the perspective for developing countries is similar. For example, Odekunle et al [50] reported for Sub-Saharan Africa the same vision as it was uncovered in our review. EHRs will improve quality of care in Sub-Saharan Africa, but high costs of procurement and maintenance of the EHR system hindered their widespread adoption until now.

### Comparison With Prior Work

In 1963 the then American President, John F. Kennedy, was pointed to the potential of health record systems: “The application of computer technology to the recording, storage, and analysis of data collected in the course of observing and treating large numbers of ill people promises to advance our understanding of the cause, course, and control of disease” [51]. Forty-five years later, another American President (in 2009) proposed a fundamental change to the use of IT in the national health care system by passing the HITECH Act [52]. Besides other regulations, each person in the United States should have an EHR by 2014 [53]. With the idea of a meaningful use, health care providers and hospitals should be rewarded for using an EHR under the Medicare and Medicaid schedule. The time gap between expectations and routine application makes it clear that the proposed advantages were neither easy to demonstrate nor easy to achieve [54]. Even a proposal in 1991 for a nationwide implementation of electronic records in the next decade failed [14]. Whether an evaluation of a technology in one country could be transferred to another one remains questionable, considering different health care systems and different strategies implemented with regard to the digitization of health care [55].

Our result of the positive impact of EMRs on the quality of care is supported by a systematic review by Campanella et al [56]. Their meta-analysis of 47 studies revealed a reduction of documentation time, a higher guideline adherence, and a lower number of medication errors and adverse drug events in the intervention group using an EHR. However, no association with mortality was found. Different to our review, the authors included studies on CPOE and did not focus on a specific area. The effect on mortality might be too small to be statistically significant even in a meta-analysis. Therefore, the inclusion of secondary data studies in our review series was reasonable. Thompson et al [57] also did not find a positive impact of EMRs on mortality. Besides, they did not find a positive impact on length of stay and costs. Their results were similar for record systems, CPOE, clinical decision support systems (CDSSs), and surveillance systems. In contrast to our results, Thompson et al [57] concluded that there “is not enough evidence to confidently state that electronic interventions have the ability to achieve the goal of improving quality and safety”. Moja et al [58] also did not find effects of CDSSs on mortality in their meta-analysis based on 16 randomized controlled trials [58]. The authors stated, “most of the studies were underpowered and too short to prove or exclude an effect on mortality, and effects as large as a 25% increase or reduction could still be possible.” In this day and age, where digitization is anywhere, it could become difficult to fill this gap with randomized controlled trials about EMRs using an appropriate control group. Besides secondary data analyses, ecological analysis might be worthwhile, even though the risk of an ecological fallacy exists [59]. With regard to CPOE as another subfunctionality of an EMR, Page et al [60] analyzed the evidence concerning a positive impact of quality of care. Defining a period overlapping with our study, 2000-2016, they included 23 studies with a control group. About half of the studies reported beneficial effects. However, the authors did not clearly distinguish between the effects of medication prescribing alerts as intervention and CPOE systems as infrastructure.

In summary, the impact of EMR subfunctionalities remains unclear in the literature. At a level beyond electronic records, the impact of health information exchange (HIE) as “the electronic transfer of patient data and health information between health care providers” is discussed [61]. Having EMRs as the condition, the exchange of data via HIE might bring the breakthrough in terms of quality of care and cost reduction. In their recent review, Sadoughi et al [62] considered 32 studies published between 2005 and 2016 that analyzed the financial or clinical impact of HIE. In that review, studies on EMRs were explicitly excluded. The majority of the studies were conducted in the United States (28/32), which is similar to our results. Furthermore, 19 studies were labeled as cohort studies, supporting our observation of a rather small number of controlled trials. Nearly all studies analyzing an improvement of quality showed a positive impact (16/17, 94%); 15/19 (79%) respective studies showed a positive effect on cost-effectiveness. With a similar span, these results from Sadoughi et al [62] match our review, with 78% of studies demonstrating an increase in quality of care and 56% demonstrating a reduction of costs. Contrary to a review including studies between 2003 and 2014 [63], Sadoughi et al [62] revealed a considerable progress in the use of HIE.

However, the advantages of EMRs have to be balanced with risks that are linked to IT not necessarily considered in evaluation studies. The relationship between the level of digitization and effects on quality and costs of care must not be linear. Higher levels of digitization might be correlated with higher risks that could lead to a reversion of the effect, as indicated by a study about the HITECH Act [64]. Therefore, it might be worthwhile to focus on the appropriate level of health IT instead of looking for global effects. Furthermore, the type of technology might not make the difference but rather the usability of the technology. For example, Roman et al [65] analyzed navigation-related issues in the field of EHRs. A lack in usability could induce risks for health care that lower the provided level of care. Finally, one should not forget that software, hardware, or electrical power supply can fail or can be a target for criminal attacks [66]. An overall perspective on the value of EMRs must therefore include a broader definition of assets and drawbacks.

### Conclusions

Our literature review revealed a clear evidence about the value of EMRs. Only some primary data studies failed to demonstrate a reduction of costs after the implementation of an EMR. Quality of care improved in all respective studies. In comparison with our first review covering the period between 1996 and 2004, the picture changed completely. At that point, only 4 of 20 studies published benefits for the quality of care and 19 reported a reduction of costs. In parallel with the appearance of the first secondary data studies, the proportions turned around in the second review from 2004 to 2010. Interestingly, the positive effects on costs could not be completely confirmed by primary data studies now. To promote an extended use of EMRs, there must be a financial refund of additional costs, given the current scientific evidence. The switch from interventional studies to observational studies using publicly available data might have induced a bias in confirming everyday perceptions about electronic records in health care. Broader and better designed studies are needed to establish better scientific evidence regarding benefits of EMRs in hospital care. Nevertheless, further studies could focus on specific aspects of electronic records to guide their implementation and operation.

## Appendix

Multimedia Appendix 1 Included studies with scoring results.

## Abbreviations

ACA: Affordable Care Act
AHA: American Hospital Association
CDSS: computer decision support systems
CMS: Centers for Medicare & Medicaid Services
CPOE: computerized physician order entry
EHR: electronic health record
EMR: electronic medical record
EPR: electronic patient record
GWTG-Stroke: Get With the Guidelines-Stroke
HCUP KID: Healthcare Cost and Utilization Project Kids Inpatient Data
HIE: health information exchange
HIMSS: Healthcare Information and Management Systems Society
HITECH: Health Information Technology for Economic and Clinical Health
HQA: Hospital Quality Alliance database
IOM: Institute of Medicine
IT: information technology
NDNQI: National Database of Nursing Quality Indicators
NIS: nursing information system
OSHPD: Office of Statewide Health Planning and Development
PDMS: patient data management system
RCT: randomized controlled trial
SID: State Inpatient Databases
WHO: World Health Organization
