# Supplementary material for: Value of the Electronic Medical Record for Hospital Care: Update From the Literature
Source: J Med Internet Res. 2021 Dec 23;23(12):e26323. doi: 10.2196/26323 (PMC8738989; doi:10.2196/26323)
Supplement: Multimedia Appendix 1 [file jmir_v23i12e26323_app1.docx]

***Appendix A. Included studies with scoring results.***

| **No.** | | **Reference** | **Study design** | **Formal quality of publication** | | **Number of users** | | **Implementation duration** | **Statistical evaluation** | **Final score** |
| --- | --- | --- | --- | --- | --- | --- | --- | --- | --- | --- |
| Primary data studies | | | | | | | | | | |
| 3 | | Castellanos et al [26] | 0 | 2 | | 0 | | 2 | 0 | 4 |
| 7 | | Feblowitz et al [30] | 0 | 0 | | 0 | | 2 | 2 | 4 |
| 17 | | Nakagawa et al [40] | 0 | 0 | | 2 | | 2 | 0 | 4 |
| 18 | | Schenarts et al [41] | 0 | 0 | | 2 | | 2 | 2 | 6 |
| 21 | | Xue et al [44] | 0 | 0 | | 2 | | 2 | 2 | 6 |
| 23 | | Zlabek et al [46] | 0 | 0 | | 0 | | 0 | 2 | 2 |
| Secondary data studies | | | | | | | | | | |
| 1 | Adler-Milstein et al [24] | | 1 | | 2 | | 0 | 1 | 2 | 6 |
| 2 | Adler-Milstein et al [25] | | 1 | | 2 | | 2 | 2 | 2 | 9 |
| 4 | DesRoches et al [27] | | 1 | | 2 | | 2 | 0 | 2 | 7 |
| 5 | Elnahal et al [28] | | 1 | | 2 | | 2 | 0 | 2 | 7 |
| 6 | Encinosa and Bae [29] | | 1 | | 2 | | 2 | 0 | 2 | 7 |
| 8 | Furukawa et al [31] | | 1 | | 0 | | 2 | 2 | 2 | 7 |
| 9 | Furukawa et al [32] | | 1 | | 2 | | 1 | 2 | 2 | 8 |
| 10 | Himmelstein et al [33] | | 1 | | 2 | | 2 | 2 | 2 | 9 |
| 11 | Jarvis et al [34] | | 1 | | 2 | | 2 | 0 | 2 | 7 |
| 12 | Jones et al [35] | | 1 | | 0 | | 2 | 2 | 2 | 7 |
| 13 | Joynt et al [36] | | 1 | | 2 | | 1 | 2 | 2 | 8 |
| 14 | Kazley et al [37] | | 1 | | 2 | | 1 | 0 | 2 | 6 |
| 15 | Lee et al [38] | | 1 | | 2 | | 1 | 2 | 2 | 8 |
| 16 | McCullough et al [39] | | 1 | | 2 | | 2 | 2 | 1 | 8 |
| 19 | Teufel et al [42] | | 1 | | 2 | | 2 | 0 | 2 | 7 |
| 20 | van Poelgeest et al [43] | | 1 | | 2 | | 0 | 0 | 1 | 4 |
| 22 | Yanamadala et al [45] | | 1 | | 0 | | 2 | 0 | 2 | 5 |
